# Comparison of Feature Selection Techniques for Power Amplifier Behavioral Modeling and Digital Predistortion Linearization

**DOI:** 10.3390/s21175772

**Published:** 2021-08-27

**Authors:** Abdoul Barry, Wantao Li, Juan A. Becerra, Pere L. Gilabert

**Affiliations:** 1 Department of Signal Theory and Communications, Universitat Politècnica de Catalunya (UPC)—Barcelona Tech, 08034 Barcelona, Spain; abdoul.aziz.barry@estudiantat.upc.edu (A.B.); wantao.li@upc.edu (W.L.); 2 Department of Signal Theory and Communications, Universidad de Sevilla, 41092 Sevilla, Spain; jabecerra@us.es

**Keywords:** behavioral modeling, digital predistortion linearization, dimensionality reduction, feature selection techniques, power amplifier

## Abstract

The power amplifier (PA) is the most critical subsystem in terms of linearity and power efficiency. Digital predistortion (DPD) is commonly used to mitigate nonlinearities while the PA operates at levels close to saturation, where the device presents its highest power efficiency. Since the DPD is generally based on Volterra series models, its number of coefficients is high, producing ill-conditioned and over-fitted estimations. Recently, a plethora of techniques have been independently proposed for reducing their dimensionality. This paper is devoted to presenting a fair benchmark of the most relevant order reduction techniques present in the literature categorized by the following: (i) greedy pursuits, including Orthogonal Matching Pursuit (OMP), Doubly Orthogonal Matching Pursuit (DOMP), Subspace Pursuit (SP) and Random Forest (RF); (ii) regularization techniques, including ridge regression and least absolute shrinkage and selection operator (LASSO); (iii) heuristic local search methods, including hill climbing (HC) and dynamic model sizing (DMS); and (iv) global probabilistic optimization algorithms, including simulated annealing (SA), genetic algorithms (GA) and adaptive Lipschitz optimization (adaLIPO). The comparison is carried out with modeling and linearization performance and in terms of runtime. The results show that greedy pursuits, particularly the DOMP, provide the best trade-off between execution time and linearization robustness against dimensionality reduction.

## 1. Introduction

The power amplifier is an active and power-hungry device present in every transmitter, being the cause of the main sources of nonlinear distortion. Many efforts have been devoted at characterizing its behavior at both circuit and system level. At the system level, black-boxes or behavioral models are used to characterize its nonlinear behavior from simple input-output data observations. In addition, when targeting PA linearization based on digital predistortion, selecting a proper behavioral model is crucial for achieving good linearization performance.

In order to overcome the low power efficiency figures of linear class AB PAs when handling current orthogonal frequency division multiplexing (OFDM)-based signals with high peak-to-average power ratio (PAPR), highly efficient amplification architectures based on dynamic load or dynamic supply modulation have been proposed in the literature. One of the solutions most commonly used by industry is the Doherty PA [1]. Alternatively, other dynamic load modulation approaches such as load modulated balanced amplifiers (LMBA) [2], LINC or outphasing PAs [3] have also been proposed. A different approach based on dynamic supply modulation, such envelope tracking PAs [4], has been adopted by the industry but mainly for mobile terminals.

The objective of these highly efficient topologies is to maximize power efficiency for high back-off levels, sometimes at the price of degrading linearity. Consequently, in order to be compliant with the linearity specifications of communication standards, for example, in terms of maximum allowed in-band and out-of-band distortion, the use of linearization techniques such as DPD becomes necessary.

When addressing the linearization of such high efficient amplification architectures, the use of multi-input behavioral models that take into account the PA dynamics (i.e., memory effects) is necessary [5]. This can ultimately impact the number of required coefficients of the PA model in order to meet the linearity specifications. Designing a DPD that includes a large number of parameters can drive overfitting. In this case, including more coefficients than the strictly required coefficients to meet the linearity specifications not only unnecessarily increases the computational complexity but may also result in an ill-conditioned least squares (LS) estimation. Consequently, a lot of research has been devoted to both reducing the number of parameters of the DPD and to guaranteeing a well-conditioned estimation [6].

In a DPD linearization system it is possible to identify the following: a forward path subsystem that includes a DPD nonlinear function (where the input signal is conveniently predistorted) operating in real-time; and an observation path subsystem where the parameters of the DPD in the forward path are estimated and updated. The coefficient update does not require real-time operation. Consequently, when targeting a field programmable gate array (FPGA) implementation, the DPD function in the forward path, for example, has to be designed as simple as possible (i.e., including the minimum and most relevant basis functions or coefficients) in order to save as many hardware logic resources and memory as possible. On the other hand, the adaptation of the DPD coefficients can be carried out at a slower time scale than in the forward path, but a well-conditioned estimation needs to be assured. For addressing the latter case, feature extraction techniques have been proposed in the field of DPD linearization with a double objective: to ensure a proper, well-conditioned parameter identification; and, as an alternative to LS solutions (e.g., QR-LS), to reduce the number of coefficients to be estimated in the observation path subsystem. Some examples of feature extraction techniques used in the field of PA behavioral modeling and DPD linearization are the following: principal component analysis (PCA) [7,8], partial least squares (PLS) [9], independent component analysis (ICA) [10] and canonical correlation analysis (CCA) [11]. These techniques are not aimed at reducing the number of coefficients of the DPD function in the forward path. Instead, they create a reduced set of new variables (orthogonal components) that are linear or nonlinear combinations of the original variables.

On the other hand, in order to reduce the number of basis functions—and consequently, coefficients—of the DPD in the forward path and to prevent overfitting, feature selection techniques have been proposed. It is possible to find, in the literature, several techniques oriented at selecting the most relevant basis functions for PA behavioral modeling or DPD linearization. However, most of the times these techniques are not properly compared with other model order reduction techniques, or they are compared with techniques of the same category, e.g., [12,13].

This paper pretends to provide a fair comparison of some of the most relevant dimensionality reduction techniques that have been proposed for PA behavioral modeling and DPD linearization. For this purpose, four categories of techniques have been considered: (i) greedy pursuits that consists in heuristic searches making a locally optimal choice at each stage. Greedy pursuits include OMP [14], DOMP [15], SP [16] and RF [17]. (ii) Regularization techniques based on a constrained LS identification that can be used to eliminate the less relevant basis functions. These techniques include ridge regression [18] and LASSO [19]. (iii) heuristic local search methods that move from solution to solution in the search space by applying local changes, e.g., finding the neighbors of previous solutions. These methods include HC [20] and DMS [21]. Finally, (iv) global probabilistic optimization algorithms oriented at finding the global best solution in the presence of multiple local optima of an unknown function with limited evaluations. These algorithms include SA [22], GA [13] and adaLIPO [23]. The comparison is conducted in terms of modeling and linearization performance and in terms of running time.

Therefore, the rest of this overview paper is organized as follows. Section 2 introduces the notations used for PA behavioral modeling and DPD linearization that are later used in the paper. Section 3 provides a short description of the aforementioned techniques under comparison, categorized in four families of dimensionality reduction algorithms. Experimental results comparing the modeling and linearization performance of the selected algorithms are discussed in Section 4. In addition, details on the test setup used, the specific behavioral model, the experimental data or the metrics are given in Section 4. Finally, conclusions are given in Section 5.

## 2. Principles of PA Behavioral Modeling and DPD Linearization

**Figure 1:**
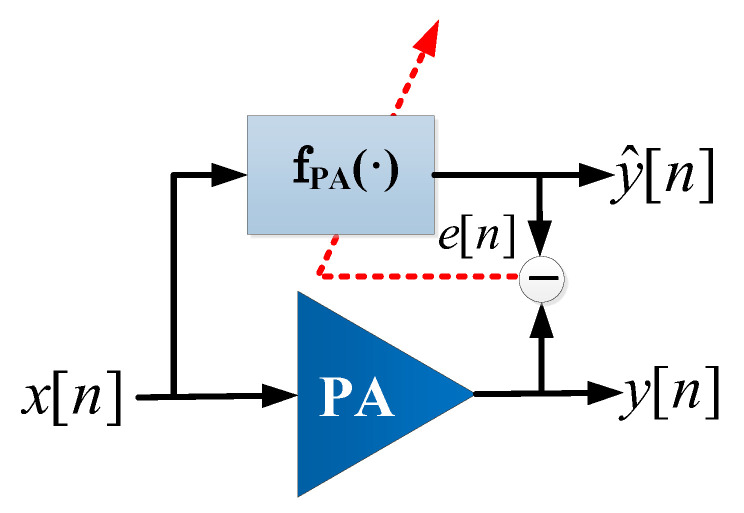
Block diagram of power amplifier (PA) behavioral modeling.

In a first approach, in order to later describe the dimensionality reduction algorithms, the notation used to describe PA behavioral modeling and DPD linearization are summarized in this section. A block diagram of PA behavioral modeling is depicted in Figure 1.

Following the notation in Figure 1, the general framework for modeling is based on the following measurement equation:

(1) y=Xw+e,

where $y={\left[y\left[n\right],y\left[n-1\right],\ldots ,y\left[n-\left(N-1\right)\right]\right]}^{T}$ is a set of measured output samples; $X={\left[\phi_{1}\left(x\right),\phi_{2}\left(x\right),\ldots ,\phi_{O}\left(x\right)\right]}^{T}$ is the model (or data) matrix that holds the basis functions $\phi_{i}$ with i=1,⋯,O; $x={\left[x\left[n\right],x\left[n-1\right],\ldots ,x\left[n-\left(N-1\right)\right]\right]}^{T}$ is the vector of input signal samples; w is the model coefficients vector; and e accounts for the modeling error. The structure of the basis functions is model dependent, and in general these consist of multiplications of delayed and conjugated versions of the PA input signal. For example, taking into account simplified versions of the general Volterra series, such as the memory polynomial (MP) [24], the generated regressors take the following form:

(2) $$\phi_{p,q}=x\left[n-q\right]{\left|x\left[n-q\right]\right|}^{p-1},$$

where *p* and *q* are the order and the memory depth of the regressor, respectively. Another widely-used model is the generalized memory polynomial (GMP) [25] that enables a shift *m* between the input signal and its envelope:

(3) $$\phi_{p,q,m}=x\left[n-q\right]{\left|x\left[n-q+m\right]\right|}^{p-1}.$$

The estimation of model coefficients can be attained through an LS regression as follows:

(4) $$\hat{w}=X^{†}y,$$

where $X^{†}={\left(X^{H}X\right)}^{-1}X^{H}$ is the Moore–Penrose pseudo-inverse and *H* stands for the Hermitian matrix. The output signal estimation follows:

(5) $$\hat{y}=f_{PA}\left(x,n\right)=X\hat{w}.$$

where $f_{PA}\left(x,n\right)$ is the nonlinear function with memory that describes the PA nonlinear behavior and its dynamics. This nonlinear function is expressed as a linear combination of nonlinear basis functions weighted by the coefficients in $\hat{w}$.

The main issue in practical applications of modeling through Volterra-based series is that the number of coefficients rapidly grow with the memory depth and order of the model. This fact, known as the curse of dimensionality, results in models that are computationally too expensive. Moreover, in the regression of these models, numerical problems arise due to the ill-conditioning of their measurement matrix.

Regressors generated by Volterra-based models are highly correlated. These similarities can be exploited with sparse signal processing, aiming at selecting the most relevant basis functions. Therefore, dimensionality reduction techniques are aimed at minimizing the number of basis functions (i.e., $\ell_{0}$-norm) subject to a constraint on the $\ell_{2}$-norm squared of the identification error. Particularizing for the identification of the PA behavioral model coefficients, the optimization problem can be described as follows.

(6) $$\begin{array}{ccc} & \min_{w}{∥w∥}_{0}\\ \mathrm{subject}\;\mathrm{to} & {∥y-Xw∥}_{2}^{2}\leq \varepsilon & \end{array}$$

Unfortunately, this is a non-deterministic polynomial-time hard (NP-hard) combinatorial search problem. Therefore, in the field of PA behavioral modeling and DPD linearization, several suboptimal approaches have been proposed, targeting both robust identification and model order reduction. Generally speaking, selection techniques provide a support set *S* that holds the indices of the relevant components. The regression of a pruned model is equivalent to (4) but only using the columns of the measurement matrix indicated by the support set. The selection of the columns of a matrix X indicated by the support set *S* is denoted by $X_{S}$ in this work.

**Figure 2:**
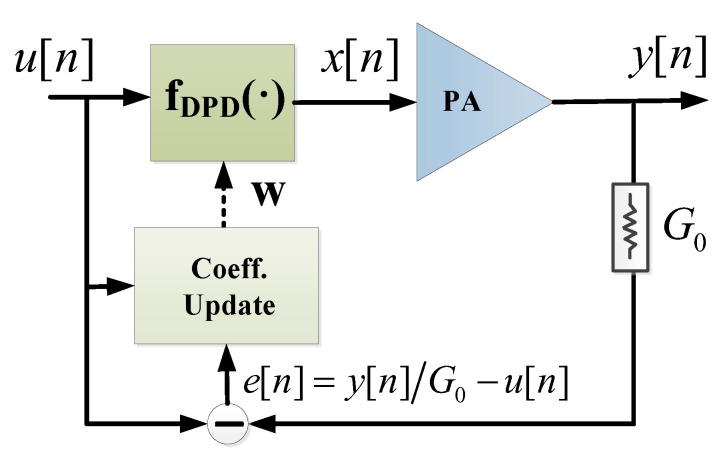
Closed-loop digital predistortion linearization: direct learning approach.

For DPD linearization purposes, an a priori study of the most relevant basis functions is conducted offline by using dimensionality reduction techniques. Then, once the data matrix containing the most relevant basis functions ($U_{S}$ in this work) for DPD linearization is determined, the coefficients of the DPD are found iteratively in a closed-loop fashion. Following the notation of the closed-loop DPD linearizer in Figure 2, the input-output relationship at the DPD block in the forward path can be described as follows:

(7) $$x=f_{DPD}\left(u,n\right)=u-U_{S}w$$

where $x={\left(x\left[n\right],\cdots ,x\left[n-1\right],\cdots ,x\left[n-\left(N-1\right)\right]\right)}^{T}$ is the predistorted data vector, $u={\left(u\left[n\right],\cdots ,u\left[n-1\right],\cdots ,u\left[n-\left(N-1\right)\right]\right)}^{T}$ the input data vector and $f_{DPD}\left(u,n\right)$ the nonlinear function with memory that describes the inverse PA nonlinear behavior. The data matrix containing the *M* most relevant basis functions for DPD linearization is defined as $U_{S}={\left[\phi_{1}\left(u\right),\phi_{2}\left(u\right),\ldots ,\phi_{M}\left(u\right)\right]}^{T}$.

Following the notation of the closed-loop adaptive DPD architecture in Figure 2, the DPD coefficients can be extracted iteratively by means of a linear LS identification as follows:

(8) $${\hat{w}}^{j+1}={\hat{w}}^{j}+\mu {\left({U_{S}}^{H}U_{S}\right)}^{-1}{U_{S}}^{H}e$$

where μ is a learning rate parameter (0<μ<1). e is the vector of the residual error defined as the following:

(9) $$e=\frac{y}{G_{0}}-u$$

where $G_{0}$ determines the desired linear gain of the PA.

## 3. Feature Selection Techniques

In the following, we provide a short description of the feature selection algorithms under comparison. These are organized in four categories. First, Section 3.1 explores greedy pursuits, and Section 3.2 covers selection techniques based on regularization. Then, Section 3.3 introduces some heuristic local search methods. Finally, techniques inspired on global probabilistic optimization algorithms are reviewed in Section 3.4.

### 3.1. Greedy Pursuits

Greedy pursuits are a kind of signal processing technique that iteratively perform a hard decision to select the most relevant coefficients of a model. When applied to the model matrix, they enable the selection of regressors by analyzing their importance, thus reducing the number of estimated coefficients.

When a behavioral model such as GMP is considered, we lack of a priori knowledge about which is the best configuration of the polynomial order, memory depth or memory of the cross-products. This results in the use of regressors that increase the complexity of the model without improving its accuracy and, eventually, results in an ill-conditioned estimation. Some of the most relevant greedy pursuits proposed in the literature oriented at addressing the optimal configuration of PA and DPD behavioral models are covered in the following sections. The specific algorithms that are analyzed are as follows: OMP, DOMP, SP and RF.

#### 3.1.1. Orthogonal Matching Pursuit

OMP is an iterative algorithm that selects the most relevant coefficient in a step-by-step manner. Each iteration selects one regressor and adds its index to the support set *S*. After the execution of the algorithm, the support set contains the indices of the coefficients sorted by their relevance.

Algorithm 1 shows the pseudocode of the OMP technique. Given a matrix of regressors X, OMP constructs the support set by adding in each iteration the regressor that has more resemblance with the residual, measured by their cross-correlation.

The input variables to this algorithm are the data matrix X and the PA output signal y. The output variables are the support set vector *S*, which contains all the regressors indexes sorted by their importance in decreasing order, and the estimated coefficient vector $\hat{w}$. Note that the indexes stored in *S* match the non-zero elements of the coefficient vector $\hat{w}$.

After initializing the residue r with a value equal to the output y, the loop starts with the calculation of the normalized correlation $g_{\left\{i\right\}}$ between the *i*-th column of the matrix of regressors, $X_{\left\{i\right\}}$, and the residue. This variable is a vector of length *M* which contains in each position a value that indicates how correlated the columns of the measurement matrix are with the residual error. Its calculation is described as follows.

(10) $$g_{\left\{i\right\}}^{\left(t\right)}\leftarrow \frac{X_{\left\{i\right\}}^{H}}{∥X_{\left\{i\right\}}{∥}_{2}}r^{\left(t-1\right)}.$$

**Algorithm 1:** Orthogonal Matching Pursuit.
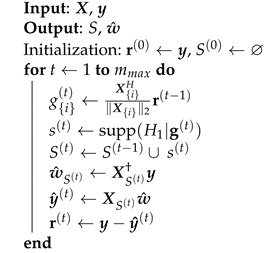


The operation $\mathrm{supp}(H_{1}|g^{\left(t\right)})$ consists of obtaining the index of the maximum value of the vector g, thus attaining the index of the most correlated element. Next, the index $s^{\left(t\right)}$ is included in the support set $S^{\left(t\right)}$. Then, an estimation of the coefficients vector with the columns that belong to the support set is performed with the following:

(11) $${\hat{w}}_{S^{\left(t\right)}}\leftarrow X_{S^{\left(t\right)}}^{†}y,$$

where the Moore–Penrose pseudoinverse $X^{†}$ computes the LS solution. The model output estimation $\hat{y}$ is obtained with the coefficients included in the support set, and finally the residual error is updated by subtracting the estimated output and the output signal.

(12) $$r^{\left(t\right)}=y-{\hat{y}}^{\left(t\right)}.$$

This last step is crucial since it eliminates the possibility of a regressor being selected more than once. The loop is repeated until the obtained residue is comparable to the precision or until the desired number of regressors is selected.

#### 3.1.2. Doubly Orthogonal Matching Pursuit

The DOMP is a variation of OMP presented in [15] to enhance the selection of model coefficients. The main difference between them is the addition of a Gram–Schmidt orthogonalization at each iteration of the OMP algorithm, decorrelating the selected regressors and those still to be selected.

The DOMP pseudocode is summarized in Algorithm 2. Similarly to the OMP, the algorithm returns a support set *S* for which its elements are sorted in decreasing impact over the output and the vector of coefficients $\hat{w}$. The initial state of the support set is empty, $S^{\left(0\right)}=⌀$, since no components have been added to it yet. Prior to the algorithm iterations, the matrix $Z^{\left(0\right)}=X$ is defined. This matrix is used to keep the information of the orthogonalized regressors, and after the algorithm execution, it will be equal to the result of applying the Gram–Schmidt procedure to the regressors matrix X in the order of the final support set $S^{\left(end\right)}$.

The algorithm performs a Gram–Schmidt orthogonalization by first obtaining the projection vector p of the selected regressor onto each one of the components of the following basis.

$$p^{\left(t\right)}=Z_{\left\{i^{\left(t\right)}\right\}}^{{\left(t-1\right)}^{H}}Z^{\left(t-1\right)}.$$

This projection is used to decorrelate the basis functions with the selected regressor as follows.

(13) $$Z^{\left(t\right)}=Z^{\left(t-1\right)}-p^{\left(t\right)}⊗Z_{\left\{i^{\left(t\right)}\right\}}^{\left(t-1\right)},$$

Hence, the selected component is orthogonal to the remaining of the basis set. Therefore, a double orthogonalization is performed as the name of the algorithm indicates. Finally, the kernel vector is computed through LS, and the residual error is updated.
**Algorithm 2:** Doubly Orthogonal Matching Pursuit.
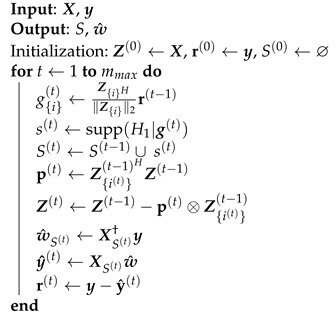


#### 3.1.3. Subspace Pursuit

DOMP and OMP are greedy pursuit algorithms that iteratively add new components to the support set, providing a sorted list of model regressors. Although these algorithms are easy to implement, they do not have recovery guarantees. SP, applied to PA model pruning in [16], is a thresholding technique which provides element selection and pruning at once with theoretical performance guarantees. Each iteration of the algorithm selects the *k* regressors with highest correlation with the residual.

The pseudocode of this technique is shown in Algorithm 3. The initialization sets the residue and the following support set.

(14) $$\begin{array}{c} r^{\left(0\right)}\leftarrow y,\; \end{array}$$

(15) $$\begin{array}{c} S^{\left(0\right)}\leftarrow ⌀. \end{array}$$

Unlike OMP and DOMP, SP does not select a single element but selects the *k* elements that exhibit the highest correlations with the residue. This operation is performed after the calculation of the following correlations:

(16) $$g_{\left\{i\right\}}^{\left(t\right)}=\frac{X_{\left\{i\right\}}^{H}}{∥X_{\left\{i\right\}}{∥}_{2}}r^{\left(t-1\right)},$$

by using the thresholding function $H_{k}\left(\cdot \right)$ that retains the highest *k* elements of its argument.

(17) $$s^{\left(t\right)}=\mathrm{supp}\left(H_{k}|g^{\left(t\right)}\right).$$

The selected components are included into the intermediate support set $S^{\left(t-0.5\right)}$. The intermediate kernel vector estimation is then performed, and the coefficients belonging to the complement of the support set are set to zero.

(18) $$\begin{array}{cc} {\hat{w}}_{S^{\left(t-0.5\right)}} & =X_{S^{\left(t-0.5\right)}}^{†}y,\\ {\hat{w}}_{\bar{S^{\left(t-0.5\right)}}} & =0 \end{array}$$

Next, the *k* highest values of ${\hat{w}}_{S^{\left(t-0.5\right)}}$ are added to the support set $S^{\left(t\right)}$. Finally, a second LS estimation is performed in order to update $\hat{w}$.

While both OMP and DOMP add one component to the support set per iteration, SP iteratively runs with a predefined desired sparsity level, providing a *k*-sparse solution until the stopping criterion is met. OMP is a particular case of SP in which k=1, since in OMP only one component is added to the support set in each iteration.
**Algorithm 3:** Subspace Pursuit.
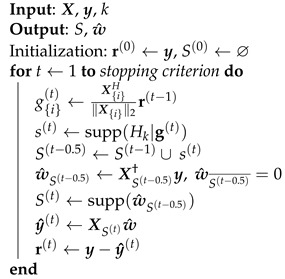


#### 3.1.4. Random Forest

RFs are an ensemble learning methods for classification or regression that operates by constructing a multitude of decision trees. The RF is capable of performing both regression and classification tasks. As its name suggests, this algorithm creates a forest with a number of decision trees. In general, the more trees in the forest, the more robust the prediction is; thus, higher accuracy can be expected.

This technique constructs a forest, i.e., a set of decision trees that are analyzed to extract the information about the relevant model regressors. This is opposed to the court model in which a single tree is built and analyzed. In order to classify a new object based on attributes, each tree provides a classification and a vote for that class. The forest chooses the classification that possesses the majority of votes over all other trees in the set. In [17], an application of the RF technique to behavioral modeling component selection was presented.

The particularization of RF for PA pruning considers binary decision trees in which the variables are flags that take the value of 0 or 1 representing the exclusion or inclusion of a regressor in the model, respectively. In order to obtain homogeneous subsets, a simple technique is to split on a variable and to measure the variance of the output when the value of this variable is fixed. The variable with the lowest output variance is considered as the next one to use as a split. Hence, variance is computed as follows:

(19) $$\mathrm{var}\left(j\right)=c_{o}\;\mathrm{var}\left(S_{o}\right)+c_{i}\;\mathrm{var}\left(S_{i}\right),$$

where $c_{i}$ is the amount of samples that belong to the subset of the output $S_{i}$, which results in the *j*-th variable being equal to *i*.

**Figure 3:**
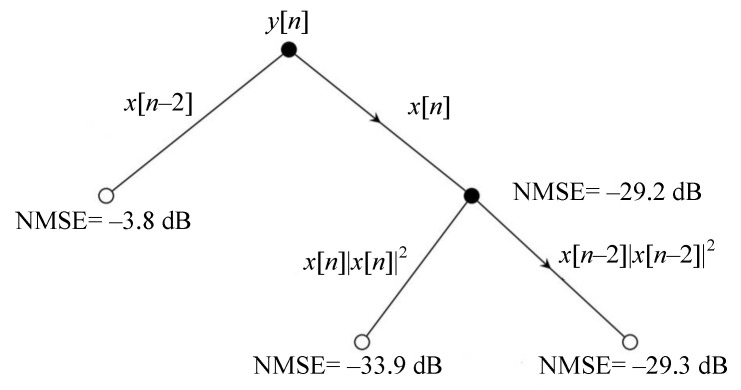
Decision tree representation of a GMP model.

When working with non-binary decision trees, a previous step needs to be performed. At binary trees, there is no need to worry about where to split the values of each variable since there are only two possible values. However, when variables are not binary, the first step requires finding the best split point at each variable. This is usually conducted by analyzing the whole range of values of the variable, dividing the output dataset and comparing the variance computed as described in (19) until the lowest is found. This process continues until the maximum number of splits, known as the depth of the tree, is reached. A graphical representation of the inclusion of GMP basis functions in a model is shown in Figure 3.

The RF code is summarized in Algorithm 4. The algorithm inputs are a set of regressors matrix X containing *M* columns and *N* rows, the output signal y and the number of trees $N_{T}$. The algorithm returns an output vector of length *M*, where each value indicates the importance of the corresponding regressor. In the first loop of the algorithm, a matrix such as the one represented in Table 1 is generated. This matrix contains a row per model, and in each of its columns has a flag that indicates the inclusion of the regressor in the model. The normalized mean square error (NMSE) of each row is calculated next.
**Algorithm 4:** Random Forest algorithm [17].
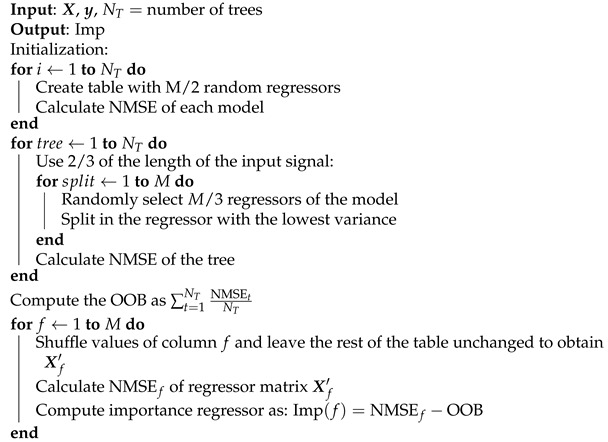


In the second loop, different decision trees are generated where each decision tree contains its determined splits by taking into account the variance. The out-of-bag (OOB) error is computed as the average NMSE of all the decision trees previously calculated. In order to compute the output vector Imp, the value of each column is modified while leaving the rest of the columns intact, and the new NMSE obtained for each case is calculated. The importance is calculated as the subtraction between the NMSE for that case and the OOB. The indexes of regressors can be ordered from highest to lowest according to the importance vector.

### 3.2. Regularization Techniques

Regularization is a process of introducing additional information in order to prevent overfitting. In general, the main idea of the regularization techniques is to add a regularization term R(w) to the cost function.

(20) $$J\left(w\right)={∥y-Xw∥}_{2}^{2}+\lambda R\left(w\right)$$

Regularization techniques are, therefore, aimed at preventing numerical instabilities due to the regression of ill-conditioned problems. In this section, regularization techniques such as ridge regression or LASSO are adapted to be used for dimensionality reduction purposes.

#### 3.2.1. Ridge Regression

In ridge regularization, the goal is to minimize the residual sum of squares subject to a constraint on the sum of squares of the coefficients.

(21) $$\begin{array}{cc} \underset{w}{min}\; & {∥y-X\cdot w∥}^{2}\\ \mathrm{subject}\;\mathrm{to}\; & \sum_{i=1}^{M}{\left|w_{i}\right|}^{2}\leq r_{2}. \end{array}$$

This restriction forces the coefficients to be in a sphere of radius $r_{2}$. An equivalent representation of the cost function can be written, particularizing $R\left(w\right)={∥w∥}_{2}^{2}$ in (20):

(22) $$\underset{w}{min}{\;∥y-X\cdot w∥}^{2}+\lambda {∥w∥}_{2}^{2},$$

where the shrinkage parameter λ acts as a tradeoff between the modeling error and the $\ell_{2}$ norm of the model coefficients. One the advantages of ridge regression is that the estimation can be written in a closed form:

(23) $$w_{\mathrm{Ridge}}={\left(X^{H}X+\lambda I\right)}^{-1}X^{H}y,$$

where I is the identity matrix. The effect of the shrinkage parameter λ on the coefficients estimate $w_{\mathrm{Ridge}}$ is to attenuate its norm as it grows. When λ tends to zero, Equation (23) tends to the LS solution. In order to attain the optimum shrinkage parameter, $w_{\mathrm{Ridge}}$ is normally calculated for different values of λ.

Based on ridge regression, a coefficient selection algorithm can be set. Algorithm 5 shows the main steps of pruning by using ridge regression. The algorithm input parameters are the normalized regressor matrix X, the vector of PA output signal y and a vector *K* that contains all the values of λ under evaluation. The algorithm provides as output a matrix W in which the columns contain the indexes of the selected coefficients by taking into account the λ vector. Note that the length of K is the same as the number of columns of W.

The ridge algorithm execution is summarized next. First, the algorithm finds a value from λ that reduces the coefficients obtained to below $w_{threshold}$ in the modulus. With this, we assure that the matrix is well regularized. Then, the indexes of coefficients that meet the condition for that particular λ are selected and stored in the output matrix W as a column. These steps are repeated for all input lambdas.
**Algorithm 5:** Sparse Ridge regression.
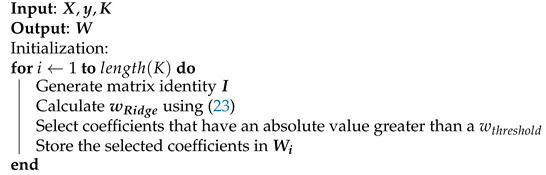


#### 3.2.2. Least Absolute Shrinkage and Selection Operator

Similarly to ridge regression, LASSO can be used for both regularization and to generate a sparse model. Whereas the constraint of the ridge regression is the sum of the $\ell_{2}$ norm of the coefficients, the LASSO constraint consists of the sum of the absolute value of the coefficients, i.e., it represents an $\ell_{1}$ norm optimization. Thus, the solution of LASSO regression satisfies the following optimization problem.

(24) $$\begin{array}{cc} \underset{w}{min}\; & {∥y-X\cdot w∥}^{2}\\ \mathrm{subject}\mathrm{to}\; & \sum_{i=1}^{M}\left|w_{i}\right|\leq r_{1}. \end{array}$$

The constrained cost function can also be written by particularizing $R\left(w\right)={∥w∥}_{1}$ in Equation (20) as follows.

(25) $$\underset{w}{min}{\;∥y-X\cdot w∥}^{2}+\lambda {∥w∥}_{1}.$$

Unlike ridge regression, LASSO has no closed form. The regression coefficients are efficiently estimated by means of the least angle regression (LARS) algorithm proposed in [26]. Since the LASSO estimation is itself a sparse solution, there is no need for selecting the highest-norm coefficients nor to set a threshold for obtaining the pruned coefficient vector, which we performed when considering the ridge regression. The pseudocode of the LASSO technique is shown in Algorithm 6.
**Algorithm 6:** Least Absolute Shrinkage and Selection Operator.
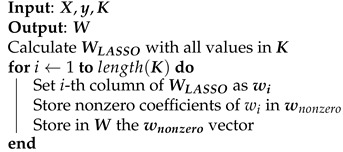


The input and output variables of LASSO are the same compared to those of the ridge regression. The explanation of these have been omitted for the sake of simplicity.

### 3.3. Heuristic Local Search Methods

#### 3.3.1. Hill Climbing Algorithm

HC is a local search technique that continuously selects the potential solution in the direction of increasing elevation in order to find the maximum of a cost function, i.e., the best solution to the problem. Its execution is terminated when it reaches peak values and no neighbor is able to enhance the cost function value. Although this family of algorithms are conceptually simple, they tend to fall into the local minima.

An algorithm based on a HC search on the GMP model structure which provides the best trade-off between modeling accuracy and its complexity was presented in [27]. Applying the previous idea to our problem, in a discrete set each node $X_{i}$ is assigned to a unique GMP model structure. The coordinate of $x_{i}$ consists of eight dimensions, $p_{a}^{i}$, $p_{b}^{i}$, $p_{c}^{i}$, $m_{a}^{i}$, $m_{b}^{i}$, $m_{c}^{i}$, $l_{b}^{i}$ and $l_{c}^{i}$, that represent the GMP configuration parameters. The value of a cost function $J_{i}$ is associated to each node $X_{i}$. A neighbor of node $X_{i}$ is defined in [27] as a node of which parameters are $p_{a}^{i}+\delta_{1}$, $p_{b}^{i}+\delta_{2}$, $p_{c}^{i}+\delta_{3}$, $m_{a}^{i}+\delta_{4}$, $m_{b}^{i}+\delta_{5}$, $m_{c}^{i}+\delta_{6}$, $l_{b}^{i}+\delta_{7}$ and $l_{c}^{i}+\delta_{8}$ where δ∈[0,±1], with only one of the parameters being different relative to zero at a same time.

The search procedure is described in Algorithm 7. As input data, the variable r is a matrix that in each row indicates the values of the parameters, and the variable $X_{k}$ is the point from which the algorithm begins analyzing the solutions. As output data, the variable $X_{b}$ indicates the best node that the algorithm has been able to find, taking into account the given cost function.
**Algorithm 7:** Hill Climbing.
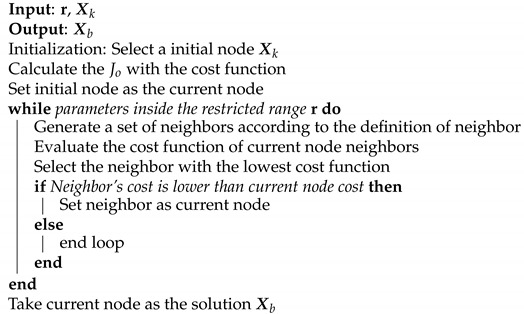


The highlights of the execution are covered next. The first step is to evaluate the cost function for the $X_{k}$ node and to set this node as the current node. Next, all the possible combinations of neighbors for that node are generated. The cost function in each of the neighboring nodes is evaluated, and the one with the lowest cost is compared with the current node. If the selected neighbor node has a lower cost than the current node, this means that better results have been found; therefore, the neighbor node is updated as the current one. In the case where the current node is the best among all its neighbors, the loop is halted, and it is returned as the optimal solution.

#### 3.3.2. Dynamic Model Sizing

DMS is a model structure adaptation algorithm proposed in [28], which can well address the challenges of adaptive model pruning problems while achieving good pruning performance. The algorithm starts from a given model structure and iteratively searches for a new model suitable for the current PA condition. In order to achieve the desired objective, the algorithm explores new basis functions that are potentially beneficial for DPD modeling and removes those that are already selected and that have a negligible impact on linearization performance. Therefore, the update of the structure mainly consists of two algorithmic steps, namely model pruning and model growing. As we can observe, this algorithm is a combination of the greedy pursuit idea of pruning the less important regressors combined with the search for other basis functions that do not belong to the initial model, which is typical of heuristic search algorithms.

The goal of the model pruning is to remove the unimportant terms in the support set in order to make the model more efficient without degrading the performance. The statistical significance of model coefficients is used as an effective and robust metric to measure the importance of model basis functions. It can be evaluated by using the *z*-test:

(26) $$\;z_{i}=\frac{|w_{i}|}{\sqrt{\nu_{i}}},$$

where $z_{i}$ is the quantification of the importance of the *i*-th regressor, $\nu_{i}$ is the *i*-th diagonal element of the inverse of the covariance matrix ${\left(X^{H}X\right)}^{-1}$ and $w_{i}$ is the LS estimation of coefficient *i*. Therefore, based on the vector Z that contains all the $z_{i}$, the importance of all coefficients in the current model can be evaluated, and the $N_{prune}$ terms with the smallest Z can be removed to attain a sparse solution.

In addition to the pruning strategy, the proposal includes a growing strategy, i.e., finding potentially important terms that are not included in the current model. A straightforward approach is to consider all possible model terms in every iteration, but this approach requires high computational complexity and can reduce the robustness of model pruning due to a large number of coefficients involved. Thus, it is desirable to consider only a subset of the full model terms that are considered useful in increasing model accuracy. The idea is to select only the neighbors of the elements that have been retained after pruning. The concept is very similar to hill climbing but with a slight difference. The idea of a neighbor is not a new model but a basis function that has the non-linear terms very close to the remaining basis function after the prune process. Taking a MP regressor as an example, the nonlinear term ${\left|x\left(n-m\right)\right|}^{\left(p-1\right)}x\left(n-m\right)$ has polynomial order *p* and memory depth *m*, so it corresponds to the point (p,m) in the feature space. Nonlinear terms that lie in its neighborhood in the feature space, such as the terms corresponding to (p±1,m) and (p,m±1), are likely to show similar modeling capabilities. Thus, new terms close to the important basis functions are added to the model during the model growing phase.

DMS instructions are given in Algorithm 8. For initialization, in order to avoid starting from scratch, the model can start from a predetermined model structure, such as a GMP model. $N_{\nu }$ is the number of new terms that is added after the process of pruning and growing at each iteration. Note that $N_{\nu }=N_{grow}-N_{prune}$; therefore, if the model structure grows by 10 and if we want to have four new basis functions added to the original set, $N_{prune}$ is going to be six. The stopping criterion set is the number coefficients; thus, the model keeps growing until it reaches a determined number of coefficients.
**Algorithm 8:** Dynamic Model Sizing.
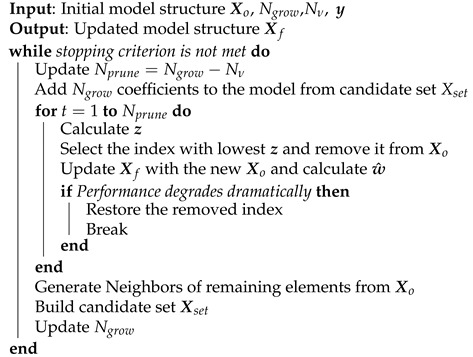


### 3.4. Global Probabilistic Optimization Algorithms

#### 3.4.1. Simulated Annealing

The SA was first introduced by Kirkpatrick in 1983 [29]. The SA method is inspired by the technique in metallurgy involving heating and controlled cooling of a material to increase the size of its crystals and to reduce their defects. It is used in many applications because ot performs well in the case of large scale searching and also has a good property of converging. The SA has also been used in the PA behavioral modeling and DPD lineariation purposes [13,23]. The SA is a modified version of the HC. Starting from a random point in the search space, a random move is made. If this move takes us to a higher point, it is accepted. If it takes us to a lower point, it is accepted only with probability p(t), where *t* is time. The function p(t) begins close to 1, but gradually reduces towards zero similar to the process of cooling a metal [30]:

(27) $$p\left(t\right)=e^{-\frac{\delta (E-E^{\prime })}{T}},$$

where $\delta (E^{\prime }-E)$ is the energy difference between current and next model to move and *T* is the temperature. If $\delta (E^{\prime }-E)$ is negative, i.e., the transition decreases the energy, the movement is accepted with probability p=1. It is important to note that the condition that the system always switches to a lower energy system when one is found is not at all necessary for the success of the method. When $\delta (E^{\prime }-E)$ is positive, the transition probability p(t) is always different from zero, i.e., the system can move to a higher energy state (worse solution) than the current state. This property prevents the system from being trapped in a local optimum. As the temperature tends to the minimum, the probability of transition to a higher energy state tends towards zero asymptotically. When *T* reaches zero, the algorithm only accepts changes to lower energy states. Due to this property, the temperature plays a very important role in the control of the evolution of the system. At high temperatures, the system tends to large energy jumps between the states, while at lower temperatures the changes in energy are smaller.

The algorithm, summarized in Algorithm 9, initiates by choosing a random model and setting the current model. The initial value of temperature *T* is an important parameter for the successful implementation of SA. If the value is too high, then it takes more reduction to converge. If too small, the search process may be less than perfect so that the points that could potentially be the global optimums are exceeded. Then, an evaluation of a new solution is performed. If $J_{i}\leq J_{c}$, then the new neighbor is accepted and it replaces $X_{c}$, updating the existing optimal solution. On the other hand, if $J_{i}\geq J_{c}$, the new neighbor $X_{i}$ can also be accepted with a probability *p*.
**Algorithm 9:** Simulated Annealing [31].
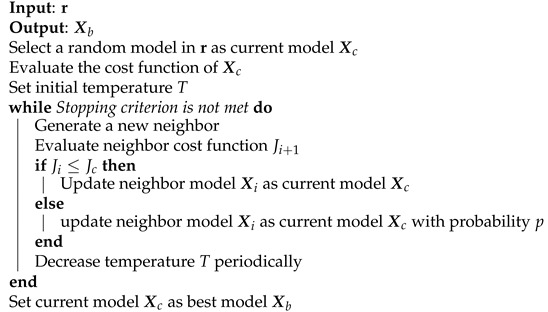


#### 3.4.2. Genetic Algorithm

GAs are based on the genetic processes of biological organisms. Over many generations, natural populations evolve according to the principles of natural selection and survival of the fittest. The individuals that are most successful in surviving have relatively larger numbers of offspring. Poorly performing individuals produce fewer or even no offspring at all.

In [13,22], GAs are used in the field of PA behavioral model to select the most relevant basis. As explained in [22], in order to perform the integer optimization, standard GAs with functions for generating integer populations and integer mutations are used. The objective is to obtain a vector of integer numbers representing the different GMP models based on a fitness function already defined.

GA deals with individuals, in this case an individual is a particular GMP model and the population is a subset of models chosen randomly from the models’ set of possible values r, as previously defined. The algorithm, summarized in Algorithm 10, applies the process of selection, crossover and mutation as the generations pass until reaching the maximum number of generations or until a stopping criterion is reached. The best individual according to the cost function is set as $X_{b}$.
**Algorithm 10:** Genetic Algorithm.
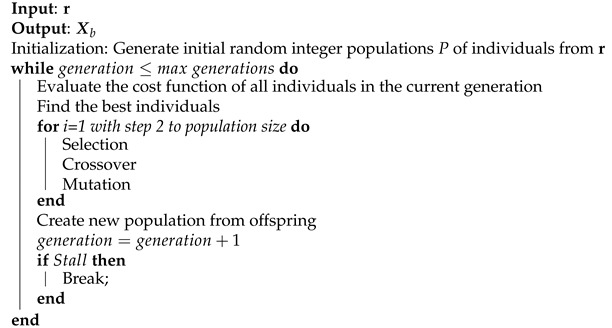


#### 3.4.3. Adaptive Lipschitz Optimization

The adaLIPO algorithm is an extension of LIPO proposed in [32], which involves an estimate of the Lipschitz constant and takes as input a parameter p∈(0,1) and a nondecreasing sequence of Lipschitz constant $k_{i\in Z}$. The algorithm, summarized in Algorithm 11, is initialized with a Lipschitz constant ${\hat{k}}_{1}$ set to 0 and alternates randomly between two distinct phases: exploration and exploitation. Indeed, at step t<n, a Bernoulli random variable $B_{t+1}$ of parameter *p*, which drives this trade-off, is sampled. If $B_{t+1}=1$, then the algorithm explores the space by evaluating the function over a point uniformly sampled over X. Otherwise, if $B_{t+1}=0$, the algorithm exploits the previous evaluations by making an iteration of the LIPO algorithm with the smallest Lipschitz constant of the sequence ${\hat{k}}_{t}$, which is associated with a subset of Lipschitz functions that probably contains *f*. Once an evaluation has been made, the Lipschitz constant ${\hat{k}}_{t}$ is updated.

The adaLIPO algorithm was used in [33] for selecting the most relevant basis functions of GMP model for DPD linearization. In order to trade off the minimization of the identification error and the number of parameters, a proper tuning of the weights defining the cost function is required.
**Algorithm 11:** Adaptive Lipschitz Optimization [32].
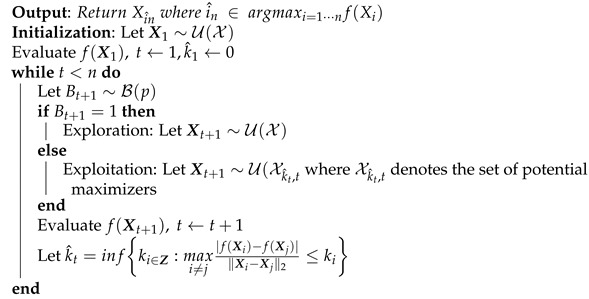


## 4. Experimental Setup and Results

### 4.1. Experimental Testbed

**Figure 4:**
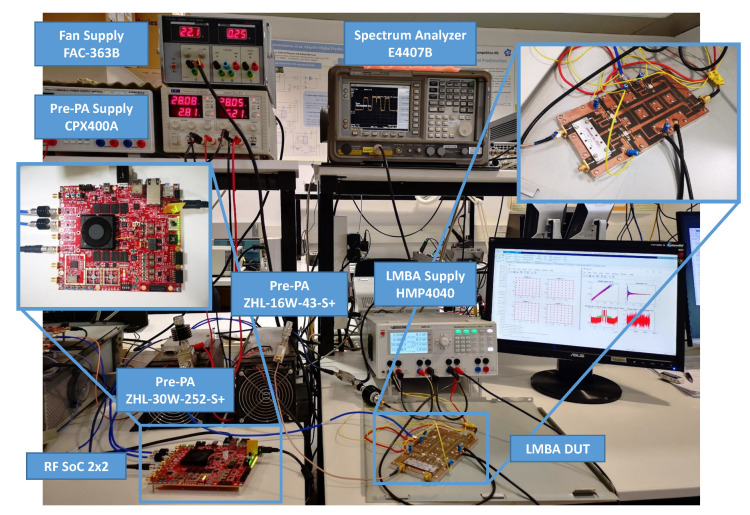
Picture of the experimental test-bed. The device under test consists in a load modulated-balanced amplifier (LMBA).

The experimental results were obtained considering the device under test relative to LMBA described in [2]. The input signals relative to the dual-input LMBA were preamplified by using two linear drivers from Minicircuits. As depicted in Figure 4, the testbed deployed to obtain experimental results comprises a software defined radio (SDR) platform for waveform playback and data capture, digital-to analog conversion (DAC), I-Q modulator and analog-to-digital conversion (ADC) for direct radio frequency sampling. The testbed is connected to a host PC, which runs as a server providing remote interface for signal generation, transmission and reception.

The SDR platform consists of a radio frequency system-on-chip (RFSoC) 2 × 2 board. The platform features a Xilinx Zynq UltraScale+ RFSoC ZU28DR FPGA device, which is supported by two 12-bit (4 GSa/s.) and two 14-bit (6.4 GSa/s.) ports. The board supports Multi-Tile Synchronization of the ADC/DAC channels; thus, we can use it to simultaneously generate the reliable RF signal and the LMBA control signal, which are phase locked and time aligned. The board uses the PYNQ framework, which is a Linux-based Python environment that eases the use of driver interfaces. We developed our custom overlay (i.e., FPGA Bitstream) to implement a pattern generator and a signal receiver that can send and receive a long signal pattern. The board can work alone or as a server. Clients (PCs) can send and receive pattern signals by using HTTP APIs. Once the pattern is loaded, the RFSoC simultaneously generates the RF signal in a cyclic mode.

For our particular validation tests, the RFSoC board was configured with a baseband sampling rate of 614.4 MSa/s. for both the ADC and the DAC tiles. In the transmission path, the baseband signals are digital up-converted and interpolated to RF signals of 4.9152 GSa/s. by the radio frequency data converter (RFDC) IP from Xilinx. In the receive path, the ADC samples, sampled at a rate of 2.4576 GSa/s, are decimated and digital down-converted by the RFDC. Since the centering LMBA is operated at a center frequency of 2 GHz, the second Nyquist zone of the ADC is used.

### 4.2. Behavioral Model, Test Data and Evaluation Metrics

The feature selection techniques presented in previous sections allow us to reduce the number of coefficients of a given behavioral model at the price of some degradation of the modeling or linearization performance. In this paper, the GMP behavioral model [25] has been considered in order to evaluate the performance of the dimensionality reduction algorithms under study. Unlike simpler behavioral models such as the MP, the GMP includes bi-dimensional kernels that take into account cross-term products between the complex signal and the lagging and leading envelope terms. This property increases the accuracy of the modeling at the price of increasing the number of coefficients. However, its complexity does not scale as quickly as the original Volterra series when considering high order kernels. Following the behavioral modeling notation, the estimated output considering a GMP can be described as follows:

(28) $$\begin{array}{cc} \hat{y}\left[n\right]= & \sum_{m=0}^{M_{a}-1}\sum_{p=0}^{P_{a}-1}\alpha_{pi}\;x\left[n-\tau_{m}^{a}\right]\;|x\left[n-\tau_{m}^{a}\right]{|}^{p}+\\ \; & \sum_{l=0}^{L_{b}-1}\sum_{m=0}^{M_{b}-1}\sum_{p=1}^{P_{b}}\beta_{pij}\;x\left[n-\tau_{m}^{b}\right]\;|x\left[n-\tau_{m}^{b}-\tau_{l}^{b^{\prime }}\right]{|}^{p}+\\ \; & \sum_{l=0}^{L_{c}-1}\sum_{m=0}^{M_{c}-1}\sum_{p=1}^{P_{c}}\gamma_{pij}\;x\left[n-\tau_{m}^{c}\right]\;|x\left[n-\tau_{m}^{c}+\tau_{l}^{c^{\prime }}\right]{|}^{p}, \end{array}$$

where $P_{a},P_{b}$ and $P_{c}$ are the nonlinearity orders of the polynomials and $M_{a},M_{b},M_{c},L_{b}$ and $L_{c}$ determine the memory and cross-memory length. $\alpha_{pi}$, $\beta_{pij}$ and $\gamma_{pij}$ are the complex coefficients describing the model, and $\tau^{a}$, $\tau^{b}$ and $\tau^{c}$ (with τ∈Z and $\tau_{0}=0$) are the most significant delays of the input signal x[n] that better contribute in characterizing memory effects. The total number of coefficients of GMP model is $O=P_{a}M_{a}+P_{b}M_{b}L_{b}+P_{c}M_{c}L_{c}$.

The initial configuration of the GMP from which the most relevant parameters are selected include the following:

- Pa=9;Pb=8;Pc=8;
- $\tau^{a}=\left[0:1:10\right];\tau^{b}=\left[0:1:10\right];\tau^{c}=\left[0:1:10\right]$;
- $\tau^{b^{\prime }}=\left[1:1:5\right];\tau^{c^{\prime }}=\left[1:1:5\right]$.

This configuration provides an initial number of coefficients of O=979.

The comparison of feature selecting techniques was carried out by considering long term evolution (LTE) (OFDM-like) waveforms. When comparing the different feature selection techniques for PA behavioral modeling, we used input-output data of the LMBA excited with a noncontiguous intra-band carrier-aggregated (CA) LTE system consisting in four channels of 64 QAM modulated LTE-20 signals (CA-4 × LTE-20) spread in 200 MHz instantaneous bandwidth at 2 GHz RF frequency and a PAPR of 10.7 dB. Different datasets were used to train and validate the PA behavioral models when considering different coefficients. Please note that although the test signal holds four channels, the model under test does not consider cross-term products of the bands but the entire bandwidth. The use of a four-dimensional model would rocket the number of coefficients to an unmanageable number, and since the single-band GMP is able to achieve a satisfactory level of linearization while still highlighting the differences between the techniques under comparison, it is considered as adequate for the experimental benchmark.

In the validation of feature selection techniques for DPD purposes, we considered the same non contiguous intra-band CA LTE system consisting of four channels of 64 QAM modulated LTE-20 signals but spread in 120 MHz. For each signal, the training dataset consisted in 307,200 complex-valued data samples, which, considering a baseband clock of 614.4 MSa/s, corresponded to 0.5 mseconds of an OFDM waveform (i.e., approximately eight OFDM symbols in LTE). The obtained LMBA configuration was later validated (including DPD linearization) by considering different batches of data of 307,200 complex-valued data samples.

In order to compare the different dimensionality reduction techniques, it is crucial to define accurate metrics to evaluate their performance. Several metrics have been proposed for both behavioral modeling and DPD linearization.

Some key metrics for PA design, such as the peak and mean output power, gain or power efficiency, are not used for the comparison of the different feature selection techniques since all of them are tested with the same DUT operated with the same configuration. However, for the sake of reference, with the LMBA used in this paper, when considering the CA-4 × LTE-20 signal with 200 MHz instantaneous bandwidth and PAPR of 10.5 dB, the linearity specifications (ACPR < −45 dBc) are met after linearization with a mean output power of around 33 dBm and a power efficiency of around 22%, as reported in [23].

For PA behavioral modeling, some of the most commonly used performance indicators are the NMSE and the adjacent channel error power ratio (ACEPR). The NMSE indicates how well the model is approximating the reality, i.e., the difference between the estimated and the real (measured) output squared, normalized by the measured output squared. The NMSE is normally expressed in dB as follows:

(29) $$\mathrm{NMSE}\;\left(\mathrm{dB}\right)=10\log_{10}\left[\frac{\sum_{n=1}^{N}|y_{meas}\left(n\right)-y_{mod}\left(n\right){|}^{2}}{\sum_{n=1}^{N}|y_{meas}\left(n\right){|}^{2}}\right],$$

where $y_{meas}\left(n\right)$ denotes the measured signal at the PA output, $y_{mod}\left(n\right)$ denotes the modeled output and N the number of samples. Since NMSE is dominated by the in-band error, it is generally used to evaluate the in-band performance of the model.

In order to highlight the out-of-band modeling capabilities, the ACEPR metric is proposed. This metric calculates power of the error between the modeled and the measured signals in the adjacent channels normalized by the in-band channel power. The ACEPR is commonly measured in dB:

(30) $$\mathrm{ACEPR}\;\left(\mathrm{dB}\right)=10\log_{10}\left[\frac{\int_{\left(adj\right)}|Y_{meas}\left(f\right)-Y_{mod}\left(f\right){|}^{2}}{\int_{ch}|Y_{meas}\left(f\right){|}^{2}}\right],$$

where $Y_{meas}\left(f\right)$ is the Fourier transform of the measured output signal and $Y_{mod}\left(f\right)$ is the Fourier transform of the modeled output signal. In the numerator, the operation is performed by taking into account the adjacent channels, while we take into account the transmission channel in the denominator.

For DPD linearization, the metrics for evaluating the in-band and out-of-band distortion are the error vector magnitude (EVM) and adjacent channel power ratio (ACPR), respectively.

The EVM is defined as the square root of the ratio between the mean error vector power to the mean reference vector power $S=\frac{1}{N}\sum_{n=1}^{N}\left(I_{ref}{\left[n\right]}^{2}+Q_{ref}{\left[n\right]}^{2}\right)$; *N* is the number of samples:

(31) $$\mathrm{EVM}\;\left(\%\right)=\sqrt{\frac{\frac{1}{N}\sum_{n=1}^{N}{\left(I_{ref}\left[n\right]-I_{meas}\left[n\right]\right)}^{2}+{\left(Q_{ref}\left[n\right]-Q_{meas}\left[n\right]\right)}^{2}}{S}}\times 100$$

where $I_{meas}$ and $Q_{meas}$ are the in-phase ($I_{meas}\left[n\right]=Re\left\{y\left[n\right]\right\}$) and quadrature ($Q_{meas}\left[n\right]=Im\left\{y\left[n\right]\right\}$) components of the measured PA output, respectively.

The expansion of the signal to the adjacent channels is characterized by the ACPR, also known as the adjacent channel leakage ratio (ACLR). The ACPR is the ratio of the power in the adjacent channels (upper and lower bands, i.e., *UB* and *LB*) to the power of the main channel (in-band, i.e., *B*):

(32) $$\mathrm{ACPR}\;\left(\mathrm{dBc}\right)=10\log_{10}\left(\frac{\int_{B}P_{out}\left(f\right)df}{\int_{LB}P_{out}\left(f\right)df+\int_{UB}P_{out}\left(f\right)df}\right)$$

Regarding the computational complexity assessment, it is common to use the Landau notation to represent the complexity (in terms of number of operations) of the algorithms. The main issue for using this figure of merit is that, for heuristic algorithms, it is very difficult to perform a complexity analysis using the Landau notation on the results. A simpler approach consists in calculating the running time of the different algorithms. Although this figure of merit strongly depends on the hardware from where the code is being executed, in this benchmark the running time is obtained by using the same hardware to make a fair comparison.

### 4.3. Experimental Results

In a first approach, a comparison of feature selection algorithms in the context of PA behavioral modeling was conducted. Therefore, despite the specific particularities of each of the previously-described algorithms it was possible to compare their performance in terms of NMSE and ACEPR versus the number of coefficients. In a second approach, after identifying some trends, a selection of the three most promising feature selection algorithms (from different families) was carried out in order to test them for DPD linearization purposes. Consequently, these algorithms were compared again, but this time in terms of key linearization indicators such as EVM and ACPR.

**Figure 5:**
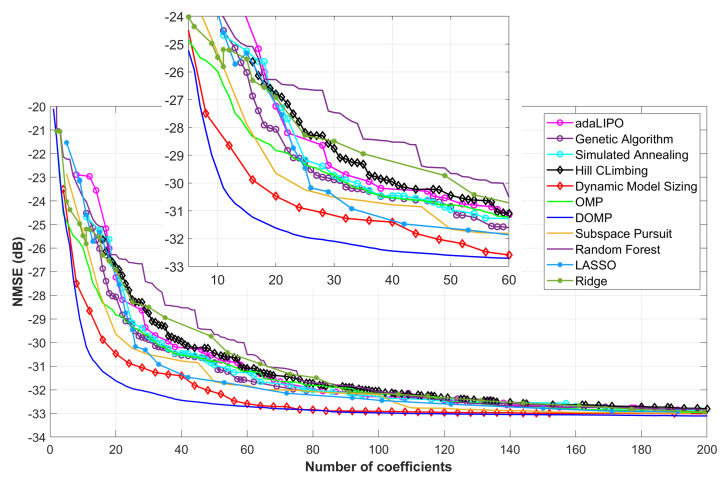
NMSE versus the number of coefficients when considering different feature selection techniques.

**Figure 6:**
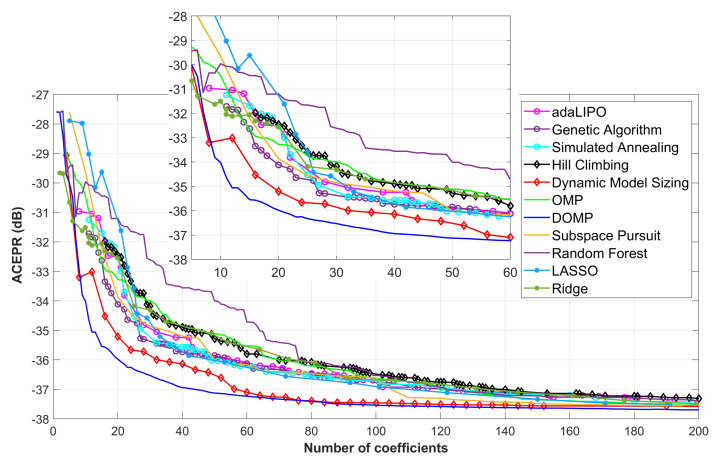
ACEPR versus the number of coefficients when considering different feature selection techniques.

Figure 5 and Figure 6 show the NMSE and ACEPR, respectively, for different number of parameters of the GMP behavioral model and by considering the aforementioned dimensionality reduction algorithms. As observed, the ones showing better NMSE and ACEPR when reducing the number of coefficients are the matching pursuit family (in particular, the DOMP and SP), the DMS and the LASSO. It is important to highlight that some of these search algorithms require tuning certain hyperparameters that determine the final results, which is the case of global probabilistic optimization algorithms (e.g., adaLIPO, SA and GA) or the heuristic local search methods (DMS and HC).

**Figure 7:**
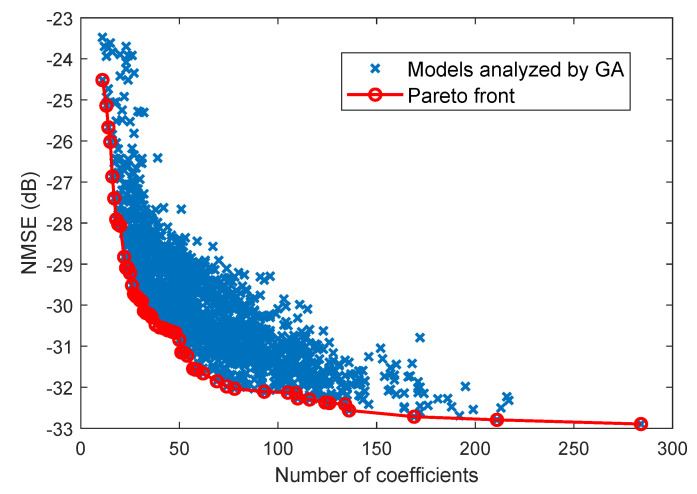
NMSE versus number of coefficients for the models analyzed in the execution of the GA and their Pareto front.

As an example, Figure 7 shows the different NMSE results obtained for the different cases evaluated by the GA. In order to include the results of this search in the comparison of Figure 5 and Figure 6, the Pareto front, which contains the best NMSE for a given number of coefficients, is extracted as depicted in Figure 7. These results were obtained after fine tuning of the hyperparameters in the fitness score, in the selection, crossover or mutation processes. It is difficult to guarantee that these are the best results that can be found, but at least they are relevant enough for showing the trend. For selecting the most suitable algorithm for DPD linearization, the necessity, or not, of tuning certain hyperparameters has to be also taken into account. For example, the regularization-based algorithms or the family of the matching pursuit do not require any critical hyperparameter tuning.

**Figure 8:**
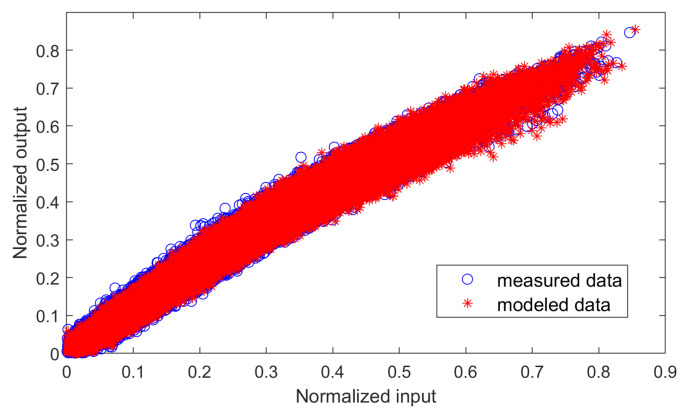
AM-AM characteristics of both the measured and modeled PA output data, considering 100 coefficients of a GMP behavioral model selected with DOMP. The resulting NMSE is −33 dB.

**Figure 9:**
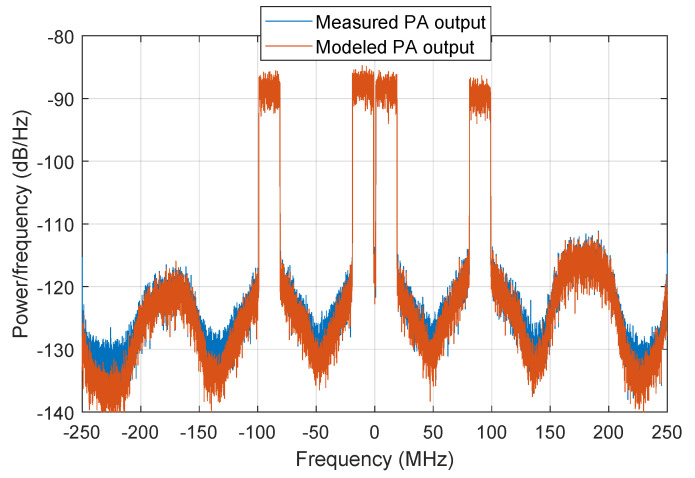
Spectra of both the measured and modeled PA output signal, considering 100 coefficients of a GMP behavioral model selected with DOMP. The resulting ACEPR is −37.5 dB.

As an example to evidence the modeling capabilities when using the GMP model with the most relevant 100 coefficients selected using the DOMP, Figure 8 and Figure 9 show the AM-AM characteristic and the spectra, respectively, for both the measured and modeled PA output data. As observed, it is possible to achieve −33 dB of NMSE and −37.5 dB of ACEPR with 100 coefficients.

**Figure 10:**
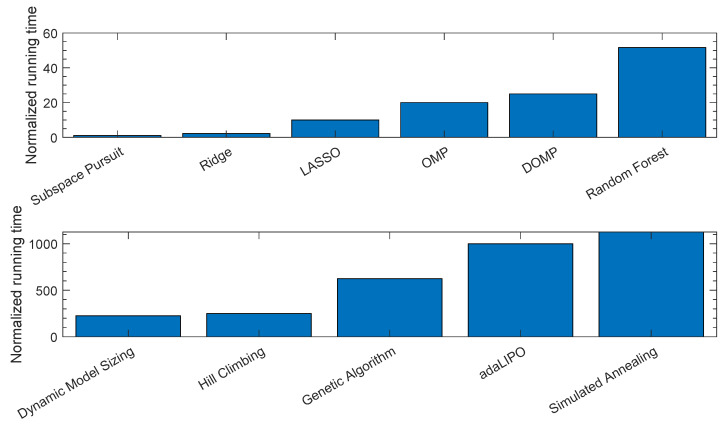
Normalized running time considering different feature selection techniques.

As discussed before, for a proper comparison of the computational complexity introduced by the algorithms, the Landau notation should be employed. However, given the difficulty of performing this study for heuristic algorithms, a comparison in relative terms taking into account the running time is provided instead. In order to obtain an idea of the order of magnitude of the running time consumed by one or another algorithm, Figure 10 shows a relative comparison by taking into account the running time of each of the algorithms normalized by the time consumed by the SP since it is the algorithm presenting the faster execution time. This comparison was obtained in the same processing hardware, by using the same training data and by considering the same initial configuration of coefficients for the greedy and regularization algorithms. Acknowledging the fact that several factors could vary the reported running time results (e.g., optimized coding, different hyperparameters and limiting the number of search iterations, etc.), the reported results in Figure 10 already provide useful information for approximately comparing the running time among the different algorithms. For example, it is quite evident that the global probabilistic optimization algorithms are the ones consuming more running time, followed by the family of the heuristic local search methods, since the number of possible combinations in the former and the possibility to explore new neighbors in the latter renders them more time consuming by nature. Instead, the family of regularization techniques or greedy pursuits exhibit a significantly lower running time since they select the most relevant ones from an original set of coefficients, which are large enough, however, to achieve good performance.

From the previous comparison of feature selection techniques in the context of PA behavioral modeling, the best three candidates were selected in order to conduct an additional comparison among them, but this time in the context of DPD linearization. These candidates are as follows: the LASSO regularization technique, the DOMP belonging to greedy pursuits and the DMS from the family of heuristic local search methods.

**Figure 11:**
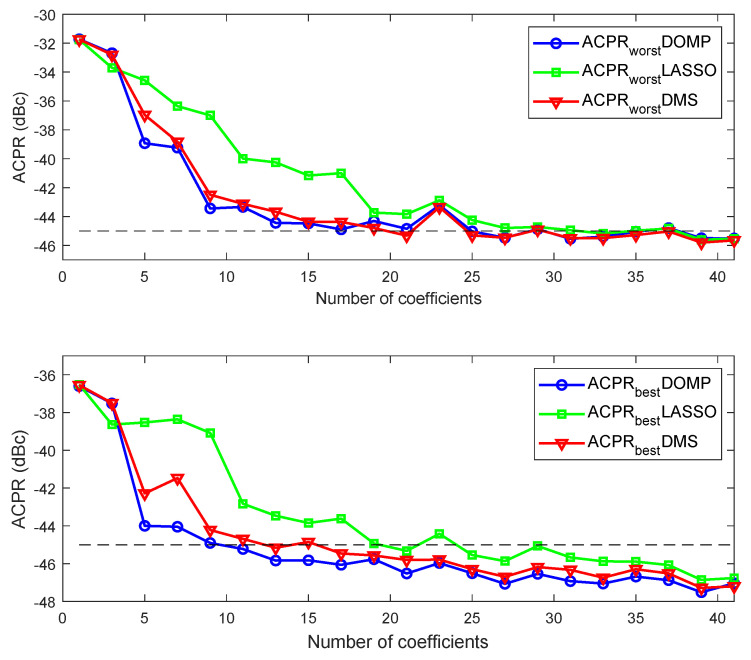
Worst (**top**) and best (**bottom**) ACPR versus the number of coefficients when considering DOMP, DMS and LASSO feature selection techniques.

**Figure 12:**
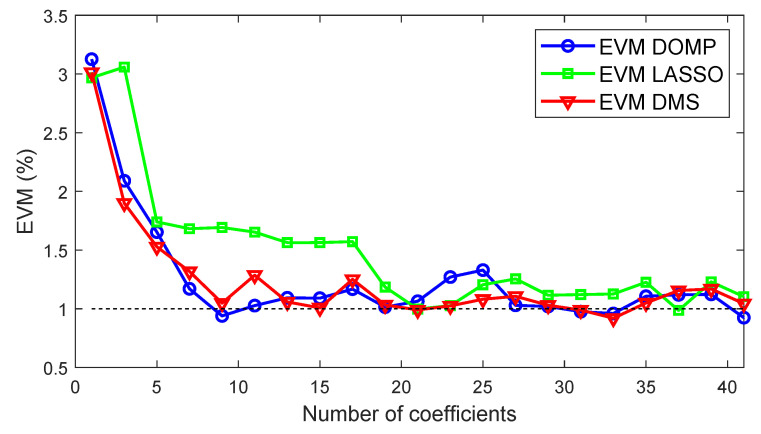
Worst EVM versus the number of coefficients when considering DOMP, DMS and LASSO feature selection techniques.

Figure 11 shows a comparison of the out-of-band distortion compensation evaluated in terms of ACPR (worst and best cases) versus the number of coefficients for the three feature selection techniques. Similarly, Figure 12 shows the shows a comparison of the in-of-band distortion compensation evaluated in terms of EVM (worst case) versus the number of coefficients. As observed, the best linearity—in both ACPR and EVM—versus the number of coefficients is obtained with DOMP, having DMS showing almost the same performance. The degradation suffered with LASSO when reducing the number of coefficients is significantly higher than with DOMP or DMS. As listed in Table 2, with only 17 properly selected coefficients using the DOMP algorithm, it is already possible to meet the out-of-band linearity specifications (i.e., ACPR < −45 dBc), while a few more coefficients are required to meet the specs with DMS. Again, with LASSO, considering 17 coefficients, the worst-case ACPR is 4 dB higher than with DOMP.

**Figure 13:**
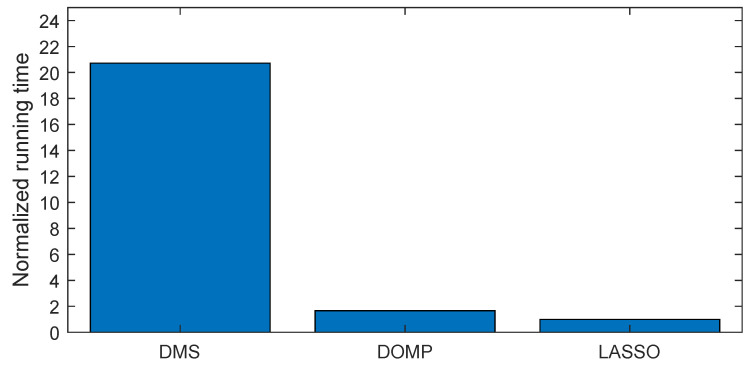
Normalized running time considering DOMP, DMS and LASSO feature selection techniques in order to select the best 200 coefficients.

Despite the DOMP and DMS showing a similar trend in linearization performance versus the number of coefficients (slightly better in the case of DOMP), when focusing in the running time, the DOMP is around 10 times faster than the DMS and around two times slower than LASSO, as shown in Figure 13. Again, the normalized running time shown in Figure 13, was obtained by using the same hardware, data and number of coefficients. However, these results can only be taken as a reference given the fact that by tuning some hyperparameters of the DMS or by optimizing some of the coding of all three algorithms, the running time may change.

**Figure 14:**
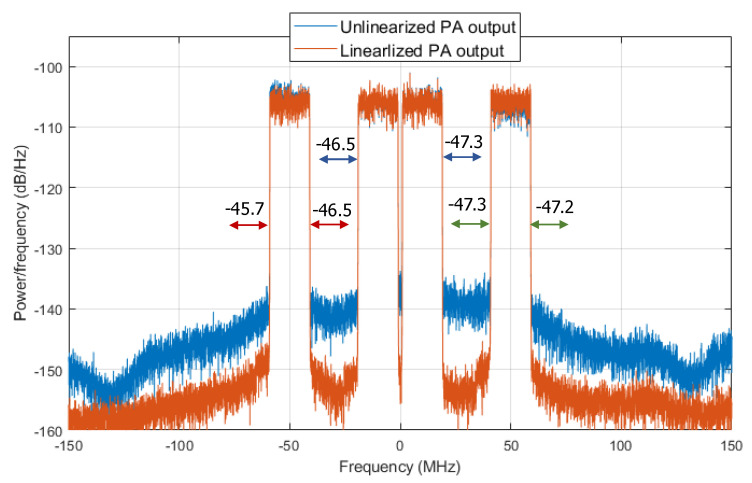
Spectra of both the linearized and unlinearized PA output signal, considering 41 coefficients of a GMP behavioral model selected with DOMP.

**Figure 15:**
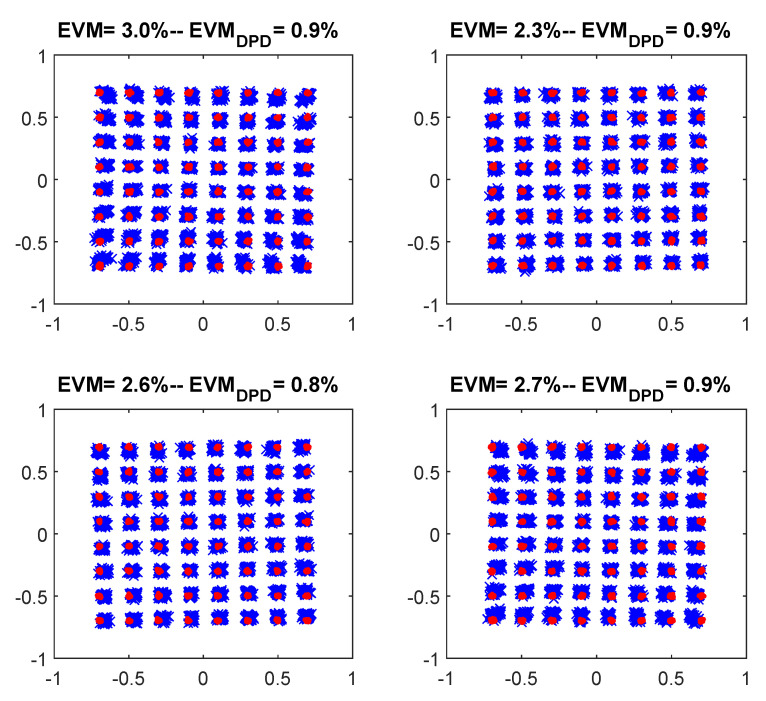
The 64-QAM constellations before and after DPD linearization, considering 41 coefficients of a GMP behavioral model selected with DOMP.

Finally, Figure 14 and Figure 15 show the output spectra and the 64-QAM constellations for each of the bands of the CA-4 × LTE-20 signal, respectively, before and after DPD linearization. The GMP DPD behavioral model used was composed by 41 coefficients after applying DOMP dimensionality reduction. The ACPR values without DPD linearization are listed in Table 2. As observed, thanks to DPD linearization, it is possible to achieve up to 14 dB of ACPR reduction and an improvement of around two percentage points of EVM.

## 5. Conclusions

In black-box modeling of the PA nonlinear behavior or DPD linearization, the use of parametric models with a significant amount of coefficients can result in overfitting or poor conditioning of the LS estimation, which ultimately degrades the modeling or linearization performance. In addition, having to deal with a huge number of coefficients results in high computational complexity and unnecessary power consumption from the digital signal processor. In this paper we have compared several dimensionality reduction methods that have been used in the field of PA behavioral modeling or DPD linearization, focusing on greedy pursuits, heuristic local search methods, regularization techniques and global probabilistic search algorithms. Each of the algorithms belonging to one of the four different categories proposed in this paper present advantages and disadvantages that have been discussed in this paper and are summarized in Table 3.

Greedy pursuits, particularly the DOMP, have shown to provide the best trade-off between running time and PA behavioral modeling or DPD linearization robustness against the reduction in the number of coefficients. In the case of DPD linearization, for example, with only 17 coefficients properly selected with the DOMP, it was already possible to meet the ACPR requirements. While regularization algorithms exhibit low computational complexity (i.e., low running time), their performance when reducing the number of coefficients is worse than with DOMP. Finally, the DMS from the family of heuristic local search methods showed similar dimensionality reduction capabilities as DOMP when properly configuring its hyperparameters (e.g, number of neighbors to grow and prune at every iteration), but at the price of requiring around 10 times more execution time. Global probabilistic search algorithms, despite being successfully used in other applications, are less suited to be used in PA behavioral modeling or DPD linearization not only because their running time is significantly higher than the rest but also because their performance depends on a proper tuning of some hyperparameters, and they do not seem to improve when compared with what can already be obtained with, for example, greedy pursuits.

## Figures and Tables

**Table 1:** Sample of input data to the RF algorithm.

*t*	$R_{1}$	$R_{2}$	⋯	$R_{m}$	NMSE
1	1	0	⋯	0	29.81 dB
2	1	0	⋯	1	22.17 dB
⋮	⋮	⋮	⋮	⋮	⋮
$N_{T}$	0	0	⋯	1	26.17 dB

**Table 2:** Linearization results obtained with a GMP DPD and considering a 4 × LTE20 signal over 120 MHz instantaneous bandwidth.

Feat. Selection	Worst ACPR	Best ACPR	Worst EVM	Best EVM	Number
Technique	(dBc)	(dBc)	(%)	(%)	Coeff.
No DPD	−31.7	−36.6	3.0	2.3	—
DOMP GMP DPD	−45.0	−46.1	1.2	0.9	17
DMS GMP DPD	−44.4	−45.5	1.3	1.0	17
LASSO GMP DPD	−41.0	−43.6	1.6	1.1	17

**Table 3:** Advantages and disadvantages of the feature selection techniques under comparison.

Category	Algor.	Advantages	Disadvantages	Best Perform.
Greedy Pursuits	OMP DOMP SP RF	List of regressors sorted by relevance Low dependence on hyperparameters tuning Moderate running time	Solutions limited to the original search space	DOMP
Regularization algorithms	Ridge LASSO	Solutions of regularized coeff. Low running time Low dependence on hyperparameters tuning	Solutions limited to the original search space	LASSO
Heuristic local search methods	HC DMS	Expands the search space by including neighbours Robust with low number of coeff.	Moderate dependence on hyperparameters tuning Significant running time	DMS
Global probabilistic search algorithms	SA GA adaLIPO	Global optimum solutions	Strong dependence on hyperparameters tuning High running time	GA

## Data Availability

The data presented in this study are available upon reasonable request from the corresponding author.
